# Identification of effector candidate genes of *Rhizoctonia solani* AG-1 IA expressed during infection in *Brachypodium distachyon*

**DOI:** 10.1038/s41598-020-71968-x

**Published:** 2020-09-10

**Authors:** Sobhy S. H. Abdelsalam, Yusuke Kouzai, Megumi Watanabe, Komaki Inoue, Hidenori Matsui, Mikihiro Yamamoto, Yuki Ichinose, Kazuhiro Toyoda, Seiji Tsuge, Keiichi Mochida, Yoshiteru Noutoshi

**Affiliations:** 1 Graduate School of Environmental and Life Science, Okayama University, Okayama, Japan; 2 Plant Pathology Department, Faculty of Agriculture, Alexandria University, El-Shatby, Egypt; 3 Bioproductivity Informatics Research Team, RIKEN Center for Sustainable Resource Science, Yokohama, Japan; 4 Kihara Institute for Biological Research, Yokohama City University, Yokohama, Japan; 5 Graduate School of Agriculture, Kyoto Prefectural University, Kyoto, Japan; 6 Institute for Plant Science and Resources (IPSR), Okayama University, Okayama, Japan

**Keywords:** Plant immunity, Plant sciences, Microbiology, Fungi, Pathogens, Transcription

## Abstract

*Rhizoctonia solani* is a necrotrophic phytopathogen belonging to basidiomycetes. It causes rice sheath blight which inflicts serious damage in rice production. The infection strategy of this pathogen remains unclear. We previously demonstrated that salicylic acid-induced immunity could block *R. solani* AG-1 IA infection in both rice and *Brachypodium distachyon*. *R. solani* may undergo biotrophic process using effector proteins to suppress host immunity before necrotrophic stage. To identify pathogen genes expressed at the early infection process, here we developed an inoculation method using *B. distachyon* which enables to sample an increased amount of semi-synchronous infection hyphae. Sixty-one *R. solani secretory effector-like protein* genes (*RsSEPGs*) were identified using in silico approach with the publicly available gene annotation of *R. solani* AG-1 IA genome and our RNA-sequencing results obtained from hyphae grown on agar medium. Expression of *RsSEPGs* was analyzed at 6, 10, 16, 24, and 32 h after inoculation by a quantitative reverse transcription-polymerase chain reaction and 52 genes could be detected at least on a single time point tested. Their expressions showed phase-specific patterns which were classified into 6 clusters. The 23 *RsSEPGs* in the cluster 1–3 and 29 *RsSEPGs* in the cluster 4–6 are expected to be involved in biotrophic and necrotrophic interactions, respectively.

## Introduction

*Rhizoctonia solani* attacks a wide range of crops and induces seed decay, seedling damping off, sheath blight, stem cankers, black scurf, and root rots^1,2^. Sheath blight disease is one of the major constraints on rice cultivation worldwide because it conduces to considerable yield losses up to 50%^3^. Highly resistant rice cultivars are unavailable for sheath blight^4^. Agrochemicals are only a practical way to suppress this disease, but caution is needed not to develop fungicide resistance^5^. To overcome this pathogen, novel crop protection measures are strongly desired which should be developed on the molecular basis of pathogen ecology and host–pathogen interaction. However, virulence mechanisms of *R. solani* are poorly understood.

Plant pathogens are categorized as biotorph, hemibiotroph, and necrotroph with their nutrition acquisition manner^6^. Biotrophs skim nutrients from living host cells, whereas necrotrophs kill host cell before or during infection and obtain nutrients from decayed tissues. Hemibiotrophs go over as biotrophs during the early infection phase and shift to necrotrophic style later on. Necrotrophs are subdivided into host-specific species and wide host-range species.

In the interaction between host and biotrophic fungal pathogens, secretory effector proteins play a pivotal role to keep down host immunity^7^. They are loaded into apoplastic space and some of them are incorporated into host cells to target pieces of machinery for enemy sensing and defense execution in plants^8^. In the studies of necrotrophic pathogens, necrosis-inducing factors including toxins and cell-wall-degrading enzymes have been in the spotlight intensively as important virulence factors. While small secreted proteins and secondary metabolites with host-specific phytotoxic activities so-called necrotrophic effectors have been identified as a critical determinant of pathogenicity of host-selective necrotrophs^9,10^. For example, PrtToxA is a small secreted protein required for pathogenicity in *Pyrenophora tritici-repentis*, a causal agent of tan spot disease of wheat^11–13^. Virulence function of these proteins needs *Tsn1* which encodes nucleotide-binding site (NBS)-leucine rich repeat (LRR)-type disease resistance (R) protein. Programmed cell death as part of hypersensitive response (HR) triggered by the recognition of pathogen-derived effector protein facilitates infection of these necrotrophic pathogens^14^. These cases are similar to the mode of action of a host-specific toxin victorin in necrotrophic fungus *Cochliobolus victoriae*, the causal agent of victoria blight disease of oat*.* It is indirectly recognized by LOV1 NBS-LRR-type R protein through thioredoxin-h5 in the host and induces disease resistance response, which makes the host susceptible^15,16^.

The importance of effector proteins in the host-nonspecific necrotrophs has recently been demonstrated as well. However, its function seems to be different from the above-mentioned cases and it is similar to those in biotrophic pathogens. *Verticillium dahliae* and *Verticillium albo-atrum* are causal pathogens of vascular wilt and tomato plants are known to exhibit race-specific resistance to these pathogens. This resistance is owing to the recognition of Ave1 effector by immune receptor Ve1^17,18^. Ave1 contributes to the virulence of this pathogen and was found in the genomes of several phytopathogens including *Fusarium oxysporum*. It was also demonstrated that a lineage-specific chitin-binding lysin motif (LysM) effector Vd2LysM in *V. dahlia* strain VdLs17, which is thought to salvage cell wall degradants working as a microbe-associated molecular pattern to stay unnoticed by the host, had a certain role in virulence on tomato^19^. A secretory protein VdSCP41 in *V. dahlia* was found to target host transcription factors CBP60g and SARD1 in *Arabidopsis thaliana* and is required for virulence on both Arabidopsis and cotton^20^.

In the case of *R. solani*, phytotoxic metabolites were explored for a long time but critical substances responsible for necrotic lesions of this pathogen have not yet been clarified^21,22^. In 2012, whole-genome sequence of *R. solani* AG-1 IA, the causal agent of rice sheath blight, was published and transcriptome analysis during the infection process was performed^23^. In this report, 965 genes encoding potential secretory proteins were predicted and expression profiles of 234 genes out of them during 10–24 h after inoculation were characterized. Then, 3 genes that encode glycosyltransferase GT family 2 domain-containing protein, cytochrome C oxidase assembly protein CtaG/cox11 domain-containing protein, and peptidase inhibitor I9 domain-containing protein were revealed to have necrosis-inducing activities when their proteins were infiltrated in not only rice but also maize. Proteomics approach has been also carried out using *R. solani* AG-8 grown in culture medium or on the wheat root for 3 or 7 days after inoculation^24^. A thaumatin protein was identified as a possible virulence factor which increased size of water-soaked disease symptom in *Nicotiana benthamiana* induced by *R. solani* infection.

We previously found that foliar treatment of salicylic acid (SA) conferred resistance to sheath blight disease caused by this pathogen in both rice and *Brachypodium distachyon*^25–27^. As a similar observation, exogenous application of benzothiadiazole (BTH), a functional SA analog, was reported to increased resistance in *Brassica napus* to *Sclerotinia sclerotium*, an Ascomycota necrotrophic pathogen^28^. These results suggest that they may go through a short biotrophic phase in its infection process which is effectively blocked by SA-dependent host defense^6^. Consistent with this scenario, we also found that particular accessions Bd3-1 and Gaz-4 of *B. distachyon* showed disease resistance against *R. solani* AG-1 IA^25^*.* Because these resistance accessions quickly induced SA-dependent marker genes after inoculation, they may recognize pathogen-derived molecules, probably effector proteins as an avirulent factor, for the induction of disease resistance response as is the case in *V. dahlia*. Taken together, *R. solani* is likely to employ effector proteins for not only pursuance of necrosis but also suppression of host immunity at the early infection stage.

Here we report the identification of effector candidate genes in *R. solani*. In combination with several bioinformatics pipelines with publicly available genome annotation and RNA-sequencing data, we extracted 61 genes as *R. solani secretory effector-like protein* genes (*RsSEPGs*). By using an improved semi-synchronous infection method of *Rhizoctonia*-*Brachypodium* pathosystem which can maximize detection sensitivity of fungi-derived transcripts especially at the early infection stage, expression profiles of 52 *RsSEPGs* during infection could be determined with a quantitative reverse transcription-polymerase chain reaction (qRT-PCR). The method could clearly distinguish their expression timing whose patterns were classified into 6 clusters.

## Results

### Modification of *R. solani* inoculation method on *B. distachyon* detached leaves

**Figure 1:**
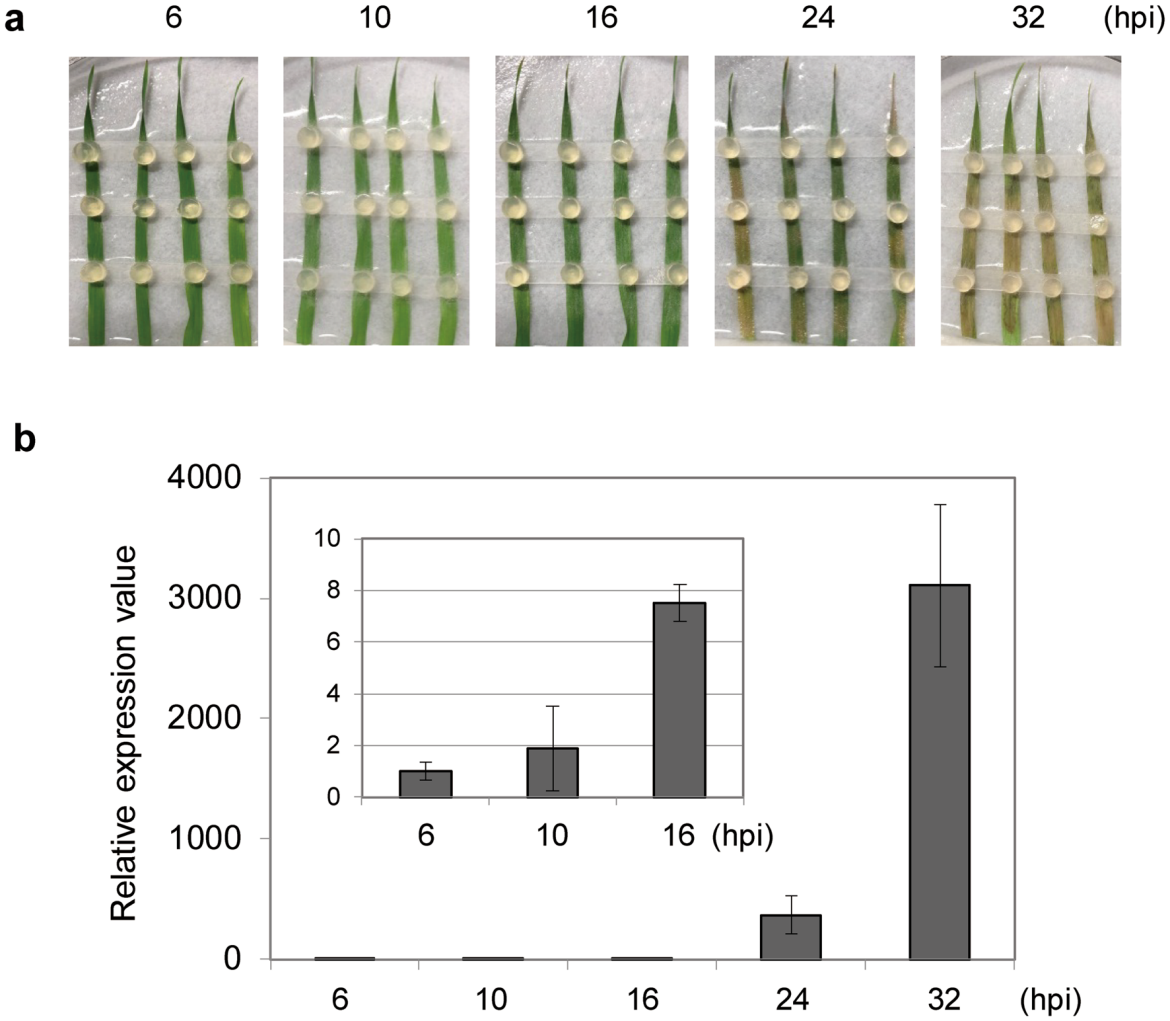
The improved inoculation method of *Rhizoctonia solani* AG-1 IA on *Brachypodium distachyon*. (**a**) Progression of infection and symptom appearance in the inoculation method. Leaves detached from 3 weeks old *B. distachyon* were placed in Petri dish with moist paper and 3 agar plugs prepared from *R. solani*-grown PDA medium were placed. The dishes were covered with lids and incubated at 25 °C. Photos were taken at the indicated hours after inoculation (hpi). (**b**) Inoculated leaves were sampled at the indicated timing and hyphae around leaves were removed before extraction of total RNAs. The relative abundance of fungal biomasses of the infected tissues was measured by qRT-PCR using cDNAs prepared from extracted RNAs with a specific primer for *18S rRNA* gene.

We have previously developed a pathosystem using *R. solani* AG-1 IA and *B. distachyon,* an experimental monocotyledonous plant^25^. To analyze gene expression profile of *R. solani* virulence genes during infection, we have carried out RNA-sequencing (RNA-seq) analysis using *B. distachyon* detached leaves inoculated with single mycelial agar plug prepared from *R. solani*-growing potato dextrose agar (PDA) medium as an inoculum (Tables S1, S2). However, the read counts derived from the fungi were so small compared with those from the host at 8 h post inoculation (hpi) and differentially expressed genes could not be fully identified with statistical significance. To overcome this technical limitation, we decided to improve our inoculation method as well as to use a qRT-PCR method for the mRNA detection. Firstly, the number of inoculums was increased from 1 to 3 on a single leaf blades to maximize amount of the infection hyphae in the samples (Fig. 1a). The synchronicity of fungal infection stage should also severely affect the result of gene expression analysis. In the case of fungal pathogen, spore inoculation meets this purpose, however, spores are unavailable in *R. solani* unfortunately because its sexual stage rarely occurs on artificial medium. Therefore, we secondary put a parafilm sheet between the inoculums and the detached leaves not to sample infection hyphae at the contact face. With this procedure, we expected enrichment of synchronous infection events from extended aerial hyphae. Thirdly, we shifted incubation temperature from 23 to 25 °C which makes the fungus more aggressive.

In this improved inoculation method, extended fungal aerial hyphae from inoculum were visibly observed at 4 hpi and the whole leaf area was covered with dense hyphae at 16 h. The timing of symptom appearance was shortened from 72 to 24 hpi compared with the previous method (Fig. 1a)^25^.

Next, infected leaves with this modified inoculation method were harvested at 2, 4, 6, 10, 16, 24, and 32 hpi, and fungal biomass in the infected leaf samples were traced by qRT-PCR using cDNAs synthesized from total RNAs with primer sets for *18S rRNA* gene^29^. Note that aerial hyphae on leaf surfaces were removed using both adhesive surgical tape and wet paper wipers with 70% ethanol. The fungal *18S rRNA* gene was reproducibly detected from 6 hpi but not 4 hpi or earlier and its level massively increased during infection up to 32 hpi (Fig. 1b).

### Evaluation of the inoculation system with characterized genes

**Figure 2:**
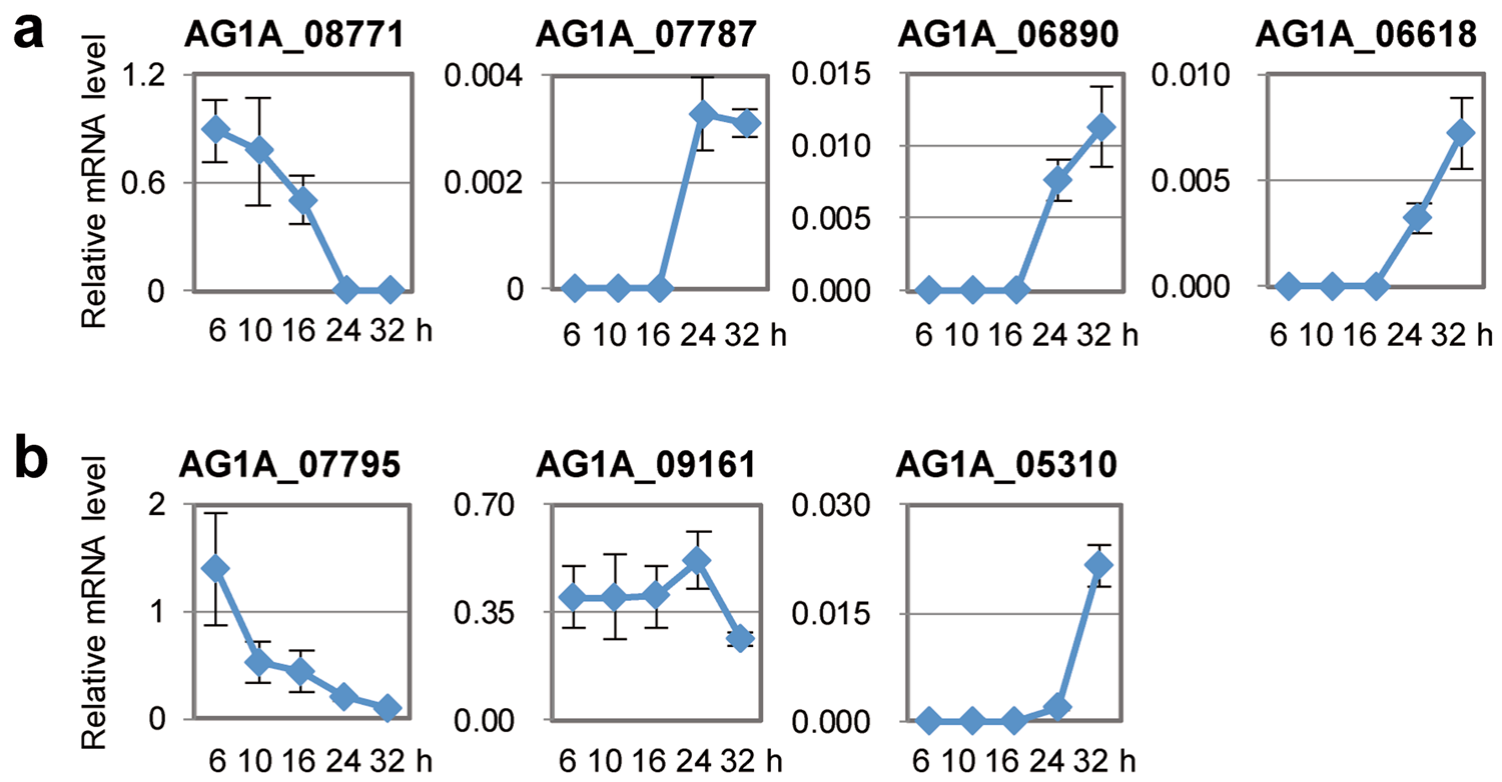
Evaluation of the inoculation method using *Rhizoctonia solani* AG-1 IA genes whose expression patterns during inoculation in rice plants have been characterized. (**a** and **b**) Expression patterns of 4 genes (*AG1IA_08771*, *AG1IA_07787*, *AG1IA_06890*, and *AG1IA_06618*) encoding carbohydrate-active enzymes (**a**) and 3 secretory effector-like protein genes (*AG1IA_07795*, *AG1IA_09161*, and *AG1IA_05310*) (**b**) of *R. solani* AG-1 IA on *Brachypodium distachyon*. Gene expressions were examined using qRT-PCR using cDNA prepared with total RNAs extracted from *R. solani*-inoculated *B. distachyon* leaves at 6, 10, 16, 24, and 32 hpi. Graphs display the relative mRNA level of each gene normalized by *18S rRNA* as an internal control. The data are presented as means with the standard error of 4 biological replicates. Experiments were performed twice with similar results and a representative result is shown.

Zheng et al.^23^ performed dual RNA-seq analysis using infected rice plants with *R. solani* AG-1 IA. Expression profiles of *R. solani* genes encoding carbohydrate-active enzymes possibly involved in fungal pathogenicity has been characterized. We selected *AG1IA_08771* (Glycoside hydrolase family 5), *AG1IA_06618* (Pectate lyase family 1), *AG1IA_06890* (Pectate lyase family1), and *AG1IA_07787* (Glycoside hydrolase family 31) and tested their expressions in our infection system. Expression of *AG1IA_08771*, which was reported to express at 18, 24, and 72 hpi, was detected with a peak at 6 hpi in our system. While, the transcriptions of the other genes, whose expression timings are at 24–72 hpi on rice, were observed at later stages such as 24 and 32 hpi in *B. distachyon* (Fig. 2a) and they were almost consistent. The 48–72 hpi in rice experiment appeared to correspond to the 24–32 hpi in the *Brachypodium* system. Next, we also examined expression profiles of secretory proteins *AG1IA_07795* (peptidase inhibitor I9 domain), *AG1IA_091651* (glycosyltransferase GT family 2 domain), and *AG1IA_05310* (cytochrome C oxidase assembly protein CtaG/cox11 domain) which could induce necrotic lesions when their purified proteins were injected into leaves of rice and maize^23^. Their transcripts were also detected during the infection on *B. distachyon* (Fig. 2b). The expression timings of *AG1IA_07795*, *AG1IA_091651*, and *AG1IA_05310* in rice infection were 18–24 (mainly at 24), 24–32 (mainly at 24), and 24–32 (mainly at 32) hpi, respectively. In our pathosystem, their expression peaks were at 6, 24, and 32 hpi, respectively. Thus, their expression profiles in rice and *Brachypodium* were also proportional basically.

### Surveillance of *R. solani* *secretory effector-likeprotein* genes (*RsSEPGs*)

Using publicly available deduced protein sequences determined by gene annotation of *R. solani* AG-1 IA^23^, we surveyed effector candidate genes. To select potential secretory proteins, SignalP 4.1 and TargetP 1.1 programs were used, and proteins possessing eukaryotic signal peptides for secretion was extracted^30,31^. To exclude membrane-localized proteins, we used TMHMM and SignalP 4.1^32^. Then, possible membrane-associated proteins with GPI-anchoring motif were detected using PredGPI algorithm, and they were removed^33^. Finally, EffectorP 1.0 program was applied to select fungal effector candidates^34^. Of total 10,541 proteins, 88 proteins satisfied the above-mentioned all criteria and they were designated as *R. solani secretory effector-like protein* genes (*RsSEPGs*) (Table S2).

### Correction of *RsSEPGs* list by calibration of gene model with RNA-sequencing data

The annotated gene model of the 88 selected *RsSEPG* was verified by RNA-sequencing data obtained from RNA extracted from *R. solani* hyphae grown on PDA medium (Table S3). Out of the total, the annotated gene models of 11 genes were found to be consistent with the RNA-seq data. Fifty-seven genes were corrected based on RNA-sequencing data. Thirteen genes have no reads detected in RNA-seq data. The remaining 7 genes have abundant transcripts in their reverse strand. Therefore, these 7 genes were removed from the list because appropriate measurements of target gene expression are thought to be prevented in further analysis.

In addition, in the above-mentioned 57 genes with calibrated annotation, 20 genes lost their secretion signal peptide in their revised gene model, therefore, they were also removed from the list. In summary, 27 genes were removed from the 88 selected *RsSEPG* and the remaining 61 genes were finally chosen as *RsSEPGs*.

### Analysis of transcriptions of *RsSEPGs* during infection in *B. distachyon*

**Figure 3:**
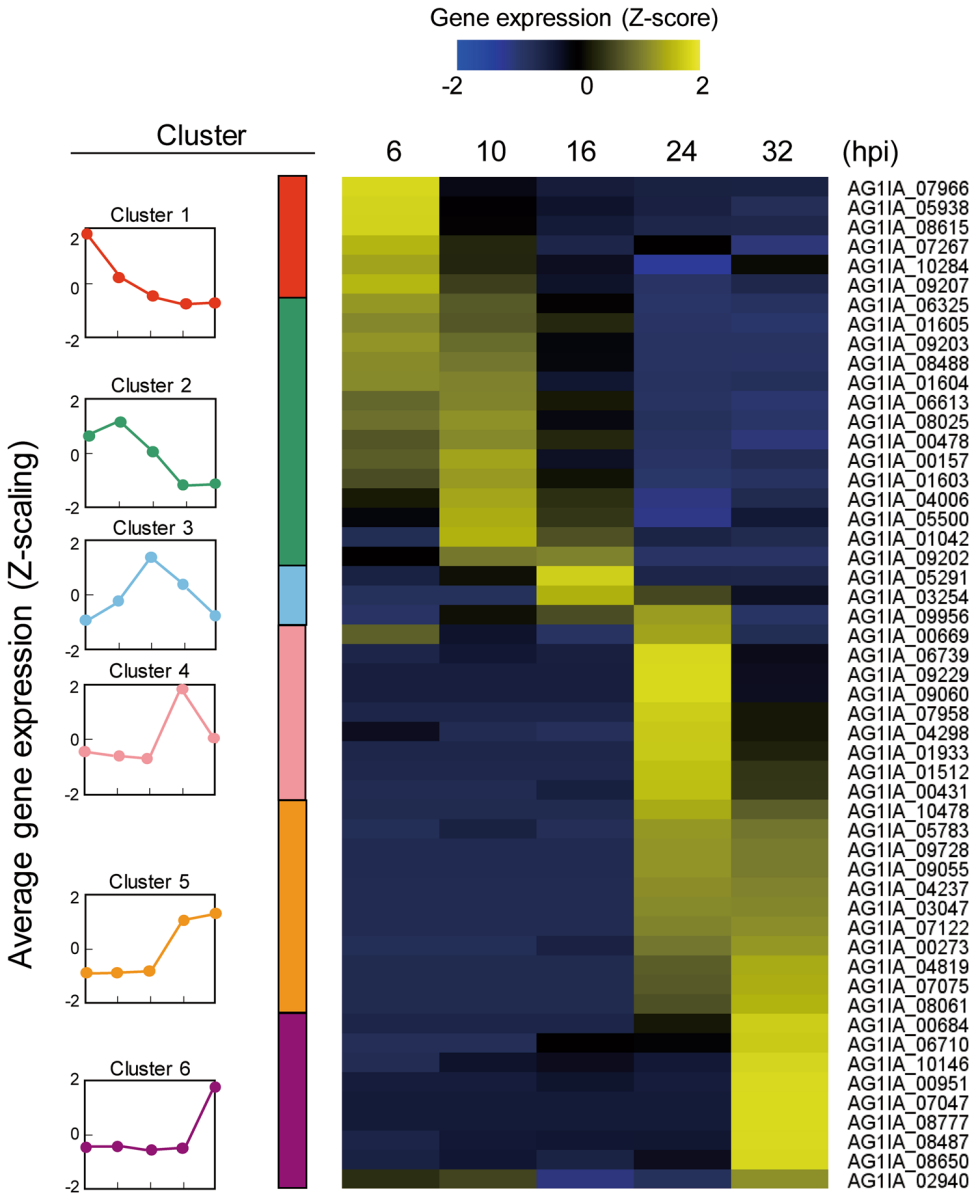
Expression dynamics and of *RsSEPGs* during the infection in *Brachypodium distachyon* and their clustering with expression patterns. Expression levels of each gene during infection (6–32 h post-inoculation) were converted to z-scores and their values were represented as a heatmap. The time point with higher expression levels compared with the others in each gene is shown with yellow color. These *RsSEPGs* were classified into 6 categories with their expression patterns by the k-means clustering method. The average pattern of each cluster was depicted with colors on the left column.

**Figure 4:**
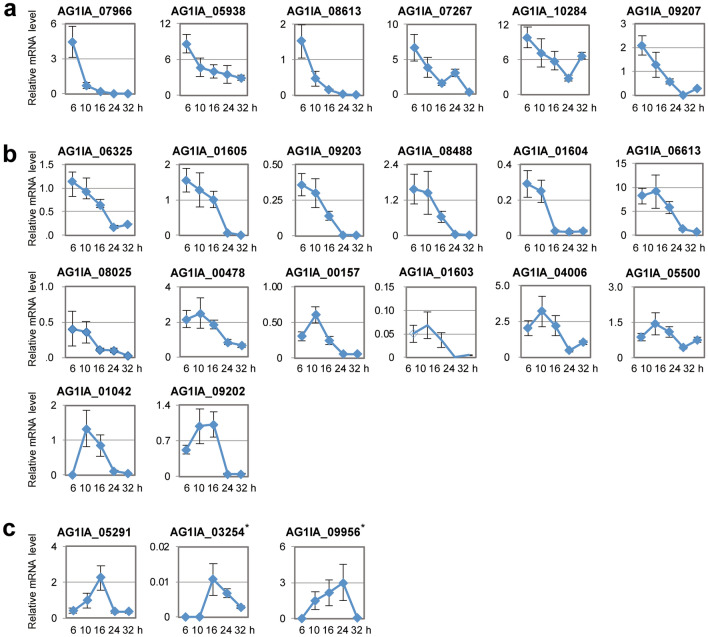
Expression profiles of *RsSEPGs* during the inoculation of *Rhizoctonia solani* AG-1 IA on detached leaves of *Brachypodium distachyon*. (**a** to **c**) Expression of 6, 14, and 3 *RsSEPGs* of *R. solani* categorized as cluster 1 (**a**), 2 (**b**), and 3 (**c**), respectively, on *B. distachyon* at 6, 10, 16, 24, and 32 h post-inoculation with the improved method. Gene expressions were examined using qRT-PCR using cDNA prepared with total RNAs extracted from *R. solani*-inoculated *B. distachyon* leaves at each time point. Graphs display the relative mRNA level of each gene normalized by *18S rRNA* as an internal control. The data are presented as means with the standard error of 4 biological replicates. Experiments were performed twice with similar results except *AG1IA_03254* and *AG1IA_09956* marked with asterisks and a representative result is shown.

**Figure 5:**
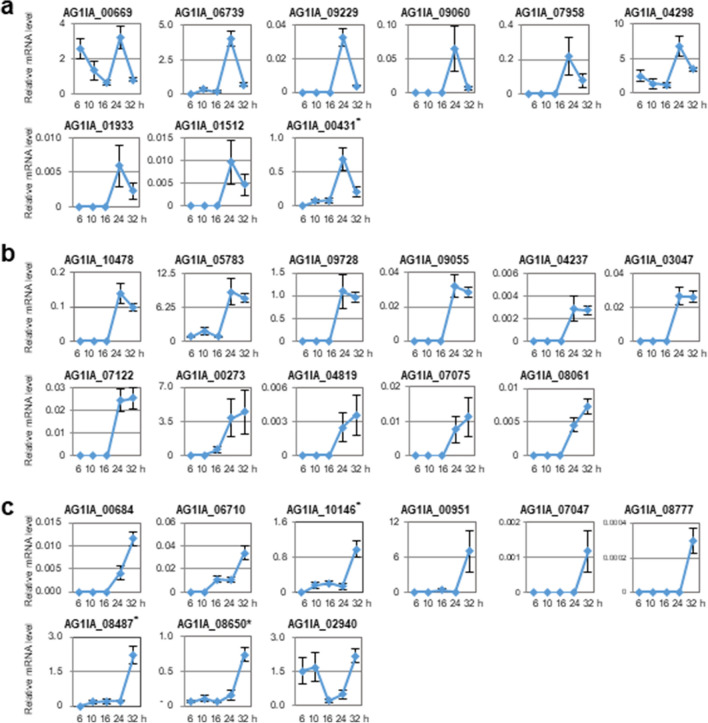
Expression profiles of *RsSEPGs* during the inoculation of *Rhizoctonia solani* AG-1 IA on detached leaves of *Brachypodium distachyon*. (**a** to **c**) Expression of 9, 11, and 9 *RsSEPGs* of *R. solani* categorized as cluster 4 (**a**), 5 (**b**), and 6 (**c**), respectively, on *B. distachyon* at 6, 10, 16, 24, and 32 h post-inoculation with the improved method. Gene expressions were examined using qRT-PCR using cDNA prepared with total RNAs extracted from *R. solani*-inoculated *B. distachyon* leaves at each time point. Graphs display the relative mRNA level of each gene normalized by *18S rRNA* as an internal control. The data are presented as means with the standard error of 4 biological replicates. Experiments were performed twice with similar results except for *AG1IA_00431*, *AG1IA_08487*, *AG1IA_08650*, and *AG1IA_10146* marked with asterisks and a representative result is shown.

To analyze expression levels of *RsSEPGs* by qRT-PCR, specific primers for each gene were designed by using the above mentioned revised gene model (Table S4). Note that annotated gene models were used for primer design in 13 *RsSEPGs* whose RNA-sequencing reads were not observed in our sample. Expression levels of *RsSEPGs* were determined as an average of 4 biological replicates (Figs. 3, 4 and 5). Transcripts of 52 *RsSEPGs* but not the others could be detected at least at a single time point tested in our experimental condition.

We performed this experiment twice and similar expression patterns could be observed except 6 genes *AG1IA_00431*, *AG1IA_03254*, *AG1IA_08487*, *AG1IA_08650*, *AG1IA_09956*, and *AG1IA_10146*, which demonstrated different expression patterns (Fig. S1).

Expressions of 9 *RsSEPGs* were not detected in this study and their transcripts were also almost undetected in PDA medium-grown hyphae analyzed by RNA-seq. On the contrary, 9 genes were expressed during infection although they were not transcribed on PDA medium (FPKM < 1) (Table S3).

### k-means clustering of gene expression patterns of *RsSEPGs*

*RsSEPGs* displayed variable expression patterns over the time course. To classify *RsSEPGs* with their expression patterns, we transformed the expression level of each *RsSEPGs* during infection into z-scores as a method for standardization and normalization which represents a variation of relative expression levels and they were depicted as a heatmap (Fig. 3)^35^. By using the k-means algorithm, the z-scaling expression profiles of 52 *RsSEPGs* could be classified into 6 clusters (Fig. 3 and Table S3)^36^. Note that, for 6 genes with varied expression patterns in two independent experiments, every single result was used as representative.

Cluster 1 includes 6 genes expressed with peaks at 6 hpi and then they were gradually decreased during infection. Cluster 2, 3, and 4 consist of 14, 3, and 9 genes, respectively, whose transcripts were predominantly accumulated at 10, 16, and 24 hpi. Transcriptions of 11 genes in cluster 5 were detected in both 24 and 32 hpi. Nine genes in cluster 6 predominantly expressed at 32 hpi. Because necrotic lesions appeared from 24 hpi in our inoculation system, the 23 *RsSEPGs* in the cluster 1, 2, and 3 are expected to have biological roles for the establishment of potential biotrophic invasion of *R. solani*. On the other hand, the remaining 29 genes in the other clusters may function mainly for the necrotic stage.

### Comparison of the expression profiles of *RsSEPGs* with the previously characterized *R. solani* secretory genes in rice

Zheng et al.^23^ identified 965 potentially secreted proteins and 234 of them were demonstrated to show differential expression with twofold or greater during early infection progress at 10–24 hpi in rice. Among 61 *RsSEPGs* identified, 23 genes are found in the list of 234 genes. Thirteen of these shared genes (*AG1IA_00273, AG1IA_00684*, *AG1IA_03254*, *AG1IA_06739, AG1IA_07047*, *AG1IA_07958*, *AG1IA_07966*, *AG1IA_08061*, *AG1IA_04237, AG1IA_08487*, *AG1IA_08615*, *AG1IA_08650*, and *AG1IA_08777*) exhibited a better correlation with similar profiles in both studies (Table S3). However, the other 5 genes (*AG1IA_00157*, *AG1IA_00669*, *AG1IA_05291*, *AG1IA_08488,* and *AG1IA_09207*) showed varied expression patterns in both studies (Table S3). Expression of 9 *RsSEPGs* was not observed in our experimental condition, but 5 of them were detected in Zheng et al.^23^ (Table S3). While 34 out of 52 expressed *RsSEPGs* were undetected by Zheng et al.^23^, 13 of them were detected in the previous alternative transcriptome analysis by Xia et al.^29^. Thus, the expression patterns of 22 effector-like genes were newly characterized in this study.

## Discussion

Transcriptome analysis during infection can be an approach for the identification of pathogen’s genes involved in pathogenicity. However, the detection of pathogen-derived transcripts using dual RNA-seq is technically problematic especially at the early infection stage due to the small amount of pathogen’s biomass in the sample. Several ingenious methods for detection or sampling have been reported so far to overcome this issue^37,38^. In this study, we simply increased the number of inoculum and restricted leaf areas for infection. Also, qRT-PCR was used as a detection method for transcripts in expectation of higher sensitivity. For this purpose, control of synchronicity of infection should have a great effect on the result. A plant sample of the broad infected area with a single inoculum should contains varied infection stages from extended aerial hyphae. In our improved inoculation method, extended aerial hypha quickly overlays small open area of detached leaf blades, therefore, it may enable to concentrate synchronous infection event as much as possible. The controlled high humidity condition with covered Petri dish also must contribute to stable and uniform infection.

By using this method, the transcripts of 52 *RsSEPGs* could be detected by qPCR at least on a single time point during the time course, and their expression patterns were characterized. Among them, expressions of 22 and 18 genes were detected in the previous dual-transcriptome analyses during infection of *R. solani* in rice by Zheng et al., 2013 and Xia et al., 2018, respectively (Table S3)^23,29^. This means that *R. solani* should use a similar set of genes for infection in the different hosts. In this study, 22 genes were newly detected, suggesting that the improved inoculation method may function well for the detection of *R. solani* RNAs *in planta*. In addition, the expression timing of each gene could be provided with a certain resolution and it would be helpful to speculate their virulence function. In this study, 6 *RsSEPGs* were detected with varied expression patterns in two independent experiments. The expression of these genes may be more sensitive to some environmental factors rather than infection progression.

Among the initially predicted 88 *RsSEPGs*, transcriptions of 68 genes were detected in our RNA-seq analysis and 57 genes (64.8%) need to be calibrated in their annotated gene model. Accurate annotation of secreted small proteins seems to be difficult. Candidate selection step using de novo assembled cDNAs based on RNA-seq data as performed by Yamamoto et al.^39^ will provide additional potential genes to be evaluated as effector.

The expression patterns of the 52 *RsSEPGs* during the infection were classified into 6 clusters and the clusters 1–3 include 23 genes with their peaks of expression level at 6–16 hpi. While the remaining 29 genes categorized as clusters 4–6 were expressed mainly at 24–32 hpi. These expression timing would be related to their function in pathogenicity. To speculate its virulence functions, sequence similarity to known proteins or domains are also useful. Among 61 *RsSEPGs*, 19 genes were given protein annotations or conserved domains with blastP and pfam search (Table S2).

Four *RsSEPGs* in the cluster 1–3 showed similarity to proteins with known functions. AG1IA_06325 has an identity (82.6%) with FK506 binding protein peptidylprolyl isomerase (a cyclophilin) which belongs to immunophilin family^40^. Cyclophilins are conserved protein families in animals, plants, fungi, oomycetes, and so on and they are reported to be involved in diverse biological processes. Their functions in pathogenicity were also demonstrated in *Magnaporthe oryzae*, *Botrytis cinerea*, *Cryphonectria parasitica*, and *Puccinia triticina*^41–44^. *Phellinus sulphurascens*, a basidiomycetes pathogen same as *R. solani*, highly induced a cyclophilin gene during the early stage of root colonization on conifer^45^. Because cyclophilins are involved in plant immunity, loading of pathogen’s cyclophilins into host cells may disturb defense response.

The amino acid sequences of *AG1IA_08487* and *AG1IA_08488* exhibit homology to ribonuclease (RNase)-containing proteins. RNase-like effectors in *Blumeria graminis* was recently found to induce disease susceptibility in wheat and *Nicotiana benthamiana* when it was expressed in the host plants. This effector is thought to be a pseudoenzyme and inhibits methyl jasmonate-triggered degradation of host ribosomal RNA leading to cell death through interaction with host RNases^46^. Since cell death works as a defense response to biotrophic pathogens, this action contributes to the establishment of biotrophy. *AG1IA_08488* expressed at early infection stage (6 and 10 hpi), its encoding protein may be a pseudoenzyme as well with a similar virulence function. This is consistent with our hypothesis. On the other hand, *AG1IA_08487* in clusters 3 or 6 also has a similar motif. It may retain RNase activity and degrade host RNAs to facilitate necrotrophic invasion.

An avirulent (AVR) effector AVR-Pita of *M. oryzae* encodes a putative neural zinc metalloprotease and it accumulates in particular surface area of infection hyphae which is called biotrophic interfacial complex (BIC)^47^. Although its virulence function has not yet been demonstrated, it is recognized by a disease resistance protein Pi-ta in rice, suggesting its function in pathogenicity^48^. Because AG1IA_00951 in cluster 6 shows similarity to metalloprotease, it might play a certain role in the formation of sheath blight necrotrophic lesions on *B. distachyon*.

Carbohydrate-binding proteins act as hydrolase enzymes for plant cell wall component and have a significant role in pathogenicity^49^. Many of carbohydrate-active enzymes encoded by *R. solani* genome are expressed during infection on rice^23,29^. In the *RsSEPGs*, *AG1IA_00669* and *AG1A_04819* were identified as genes encoding carbohydrate-binding domain-containing proteins. Given that they satisfy criteria for secretory effector, they may have specific virulence functions such as masking of microbe-associated molecular patterns as found in Lysin motifs (LysM)-domain containing proteins^50,51^.

*AG1IA_09055* and *AG1IA_09060* in the clusters 5 and 4, respectively, share homology with a thaumatin-like protein. This protein was identified as a secretory protein in *R. solani* AG-8. One thaumatin gene (*RsAG8_08836*) was significantly up-regulated in infected root tissue at 2 days post-infection and transient expression of this gene with agroinfiltration in *N. benthamiana* induced a necrosis^24^. These thaumatin-like protein genes probably have a role in necrotrophic interaction.

In the *RsSEPGs*, most of them have no characteristic motifs. They may contain avirulent factors that are recognized by *R. solani*-resistance *B. distachyon* accessions such as Bd3-1 and Gaz-4^25,26^. To open new avenues towards the understanding of the molecular pathogenicity of *R. solani,* we have started their functional analyses. Although gene knockout analysis is unavailable in *R. solani*, alternative approaches such as gene knockdown analysis, ectopic expression in plant cells, heterologous expression using bacterial pathogen, and identification of interactors from host plants can be employed. Ultimately, such knowledge will provide a basis for plant breeding as well as disease control strategies toward this pathogen.

## Materials and methods

### Plant and fungal materials

The *Brachypodium distachyon* accession Bd21 was obtained from the National Plant Germplasm System of USDA-ARS. Dry seeds were sterilized and germinated in a plastic Petri dish with moist filter paper. After 7 days, the seedlings were transferred to soil (Sakata Supermix-A; Sakata Seed, Yokohama, Japan) and grown for 2 weeks in a growth chamber with LED lights (LPH-350S; Nippon Medical & Chemical Instruments, Osaka, Japan) at 25 °C under a 16 h light/8 h dark photoperiod. The *Rhizoctonia solani* Japanese isolate sampled from rice symptoms of sheath blight disease (MAFF Genbank stock number MAFF305230) was obtained from NARO Genbank and cultured on potato dextrose agarose plates (PDA; BD, Franklin Lakes, NJ, USA) at 23 °C for 3 to 5 days.

### RNA-seq analysis

Total RNA was extracted from the *R. solani* AG-1 IA mycelia grown on PDA medium and colonized in *B. distachyon* leaves at 8 hpi in the previous infection method^25^ using Nucleospin RNA plant kit (Takara Bio, Kusatsu, Japan) with three biological replicates. The quality and quantity of the extracted RNA were checked using a NanoDrop OneC (Thermo Fisher Scientific, Waltham, MA, USA) and a 2,100 Bioanalyzer (Agilent, Santa Clara, CA, USA). Libraries for RNA-seq were prepared using a Truseq RNA library preparation kit (Illumina, San Diego, CA, USA) according to the manufacturer's instructions, and the prepared libraries were sequenced with Hiseq4000 (Illumina). For the medium samples, the obtained reads were mapped to the *R. solani* genome (GCA_000334115) retrieved from EnsembleFungi using TopHat v2.1.1 with Bowtie v2.2.6 as its mapping tool^52,53^. For the leaf samples, the obtained reads were firstly mapped to the *B. distachyon* reference genome (Bdistachyon_314) retrieved from Phytozome to remove the reads derived from host plants. The resulted unmapped reads were then mapped to the *R. solani* genome after discarding single reads. The number of mapped reads of each *R. solani* gene was counted and normalized by fragments per kilobase of transcript per million mapped reads (FPKM) using Cufflnks^54^.

### Inoculation test

The inoculation method described previously^25^ was modified. The third leaves of 3 weeks old plants were detached and fixed on a wet filter paper in a Petri dish. *R. solani* sub-cultured on PDA media for 3 days and mycelial agar plugs (3 mm in diameter) were prepared with a biopsy punch (BP-30F, Kai corporation, Tokyo, Japan). Three Parafilm strips (4 mm in width) (Parafilm M film, Bemis Flexible Packaging, Neenah, WI, USA) were placed evenly on a fixed *B. distachyon* leaves and agar plugs were placed on each of them. The Petri dishes were kept in a container with clear-plastic lid append with wet papers to make humid condition and the containers were put in a growth chamber at 25 °C.

### Fungal biomass and gene expression analysis

Inoculated leaves were sampled on several time intervals post-inoculation (2–32 hpi). The areal hyphae on the sampled leaves were removed by wet-wipes with 70% ethanol and adhesive tape and were immediately frozen in liquid nitrogen and stored at − 80 °C. Frozen leaf samples were crashed with 4 zirconia balls (ϕ 3 mm) using a homogenizer (MicroSmash MS-100, TOMY SEIKO, Tokyo, Japan). Total RNAs were extracted using PureLink RNA Mini Kit (Thermo Fisher Scientific, Waltham, MA, USA). Concentration and purity of extracted RNAs were checked using DS-11 Spectrophotometer (DeNovix, Wilmington, DE, USA). cDNAs were synthesized using PrimeScript RT reagent Kit with gDNA Eraser (Takara Bio, Kusatsu Japan). Luna Universal qPCR Master Mix (New England Biolabs, Ipswich, MA, USA) and LightCycler 96 Real-Time PCR System (Roche, Pleasanton, CA, USA) were used for qPCR analysis. All experiments were performed with 4 technical replicates and repeated 2 times as biological replicates. The expression data were normalized with *18S rRNA* gene as a reference. Primer3 online software tool was used to design primers for each gene (Table S3)^55^.

### Identification of *RsSEPGs*

Annotated protein sequences of *R. solani* AG-1 IA genome on the EnsemblFungi database (GCA_000334115) were used for searching sequence motifs^23^. Peptide for eukaryotic secretion signals was predicted using SignalP-4.1 and TargetP-1.1^30,31^ . For transmembrane domain search, TMHMM2.0 and SignalP-4.1 were used^32^. The GPI-anchoring motif was detected using PredGPI^33^. To obtain predictions as fungal effectors, EffectorP 1.0 was employed^34^.

### z-scaling and k-means clustering of expression profiles of *RsSEPGs*

The expression level of each *RsSEPG* normalized with *18S rRNA* gene during infection was converted to z-scores using the genescale function in the genefilter of Bioconductor R package^56^. The z-scaling expression profiles were classified into 6 clusters with a k-means algorithm using Multi Experiment Viewer (MeV)^57^.

## Supplementary information

Supplementary Table S2.

Supplementary Information.

## Data Availability

All data generated or analysed during this study are included in this published article (and its Supplementary Information files).
