# Supplementary material for: Identification of effector candidate genes of Rhizoctonia solani AG-1 IA expressed during infection in Brachypodium distachyon
Source: Sci Rep. 2020 Sep 10;10:14889. doi: 10.1038/s41598-020-71968-x (PMC7483729; doi:10.1038/s41598-020-71968-x)
Supplement: Supplementary file 2 — Supplementary Information. [file 41598_2020_71968_MOESM2_ESM.pdf]

Supplementary Information

Identification of effector candidate genes of *Rhizoctonia solani* expressed during infection on *Brachypodium distachyon*

Sobhy S. H. Abdelsalam[1,2], Yusuke Kouzai[3,4], Megumi Watanabe[1], Komaki Inoue[3], Hidenori Matsui[1], Mikihiro Yamamoto[1], Yuki Ichinose[1], Kazuhiro Toyoda[1], Seiji Tsuge[5], Keiichi Mochida[3,4,6], and Yoshiteru Noutoshi[1]*

*Corresponding author, Yoshiteru Noutoshi (noutoshi@okayama-u.ac.jp)

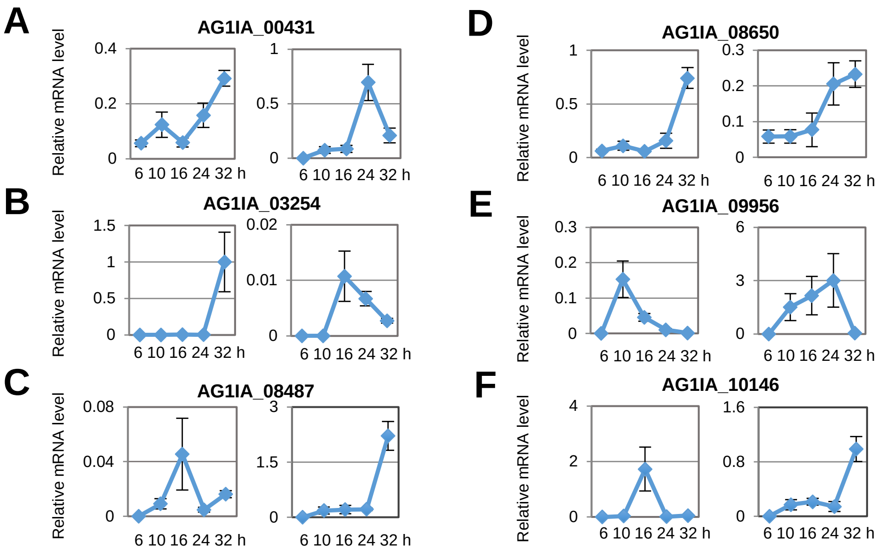

Supplementary Figure S1. Expression profiles of *RsSEPGs* during inoculation of *Rhizoctonia solani* on detached leaves of *Brachypodium distachyon* which showed different expression patterns in two independent experiments. (**A** to **F**) Expressions of *AG1A_00431* (cluster 4 or 6) (**A**), *AG1IA_03254* (cluster 6 or 3) (**B**), *AG1IA_08487* (cluster 3 or 6) (**C**), *AG1IA_08650* (cluster 6 or 5) (**D**), *AG1IA_09956* (cluster 2 or 3) (**E**), and *AG1IA_10146* (cluster 3 or 6) (**F**) of *R. solani*, which were classified into different clusters with their expression patterns, on *B. distachyon* at 6, 10, 16, 24, and 32 hours post inoculation with the improved method. Gene expressions were examined using qRT-PCR using cDNA prepared with total RNAs extracted from *R. solani*-inoculated *B. distachyon* leaves at each time point. Graphs display the relative mRNA level of each gene normalized by *18S rRNA* as internal control. The data are presented as means with standard error of 4 biological replicates. The results of two independent experiments were shown.

Supplementary Figure S1
Abdelsalam et al.

Supplementary Table S1. Summary of RNA-seq data of *Rhizoctonia solani* growing on PDA medium, and *R. solani* in detached leaves of *Brachypodium distachyon* Bd21

Sample name	Biological replicate	Mapping results to the *B. distachyon* genome					Mapping results to the *R. solani* genome				
		Number of total reads	Number of mapped reads	Mapped (%)	Properly paired (%)	Number of unmapped reads	Number of total reads	Number of mapped reads	Mapped (%)	Number of properly paired reads	Properly paired (%)
On PDA medium	1						75,820,544	62,663,857	82.65	52,149,370	68.78
	2						71,975,102	59,730,523	82.99	48,662,366	67.61
	3						74,022,804	60,630,695	81.91	50,261,484	67.90
In *B.distachyon* leaves (8 hpi)	1	51,894,736	48,761,392	93.96	85.98	3,133,344	1,812,936	36,066	1.989	30,258	1.669
	2	64,192,412	60,122,908	93.66	85.26	4,069,504	2,480,250	13,589	0.548	11,340	0.457
	3	60,687,308	56,567,307	93.21	83	4,120,001	2,586,980	11,334	0.438	9,098	0.352

Supplementary Table S3. *Rhizoctonia solani AG1IA secretory effector protein* genes (*RsSEPGs*) with their expressions during infection and agar medium

GeneID [a]	Correction of gene model	Loss of signal peptide after annotation calibration	Length (aa)	Cluster [b]	Expression timing in Zheng et al (2013) [c]	Detection of transcripts in Xia et al (2017)	Annotation [d]	Expression levels on PDA medium (FPKM) [e]			
								Rep1	Rep2	Rep3	AVG
AG1IA_00153	yes	yes	183>	n.a.	24 hpi		hypothetical protein	12.11	23.17	22.26	19.18
**AG1IA_00157**	yes	no	168	2	24 hpi		polysaccharide deacetylase domain-containing protein	4.51	7.52	9.41	7.15
AG1IA_00172 [f]	n.a.	n.a.	-	n.a.	n.a.		Cysteine proteinase	12.46	18.47	22.04	17.66
**AG1IA_00273**	yes	no	146	5	10, 18 hpi	yes	hypothetical protein	92.34	48.50	32.69	57.84
**AG1IA_00431**	no	no	232	4 or 6	n.a.		GILT domain-containing protein	215.43	168.17	173.44	185.68
**AG1IA_00478**	yes	no	90	2	n.a.		hypothetical protein	20.93	25.13	26.58	24.21
AG1IA_00529	yes	yes	160	n.a.	n.a.		hypothetical protein	8.07	6.39	7.29	7.25
**AG1IA_00669** [h]	yes	no	294>	4	10 hpi		glycosyl hydrolase family 61 domain-containing protein	91.92	140.04	126.43	119.47
**AG1IA_00684**	yes	no	67	6	10, 18 hpi		hypothetical protein	0.27	0.71	0.69	0.55
**AG1IA_00763**	n.a.	n.a.	68	n.d.	24 hpi		hypothetical protein	1.30	0.00	0.00	0.43
**AG1IA_00879**	n.a.	n.a.	156	n.d.	10 hpi		hypothetical protein	6.56	8.45	7.23	7.41
**AG1IA_00951**	yes	no	134	6	n.a.		deuterolysin metalloprotease (M35) family domain-containing protein	3107.04	2100.49	1561.70	2256.41
**AG1IA_01042**	yes	no	170	2	n.a.	yes	hypothetical protein	13.98	8.07	9.42	10.49
**AG1IA_01512**	yes	no	78	4	n.a.	yes	hypothetical protein	19.66	17.26	16.02	17.65
AG1IA_01599	yes	yes	391	n.a.	n.a.		lipoic acid synthase	86.49	86.45	91.46	88.13
**AG1IA_01603**	yes	no	85	2	n.a.		DUF1524 domain containing-protein	10.51	14.77	14.81	13.36
**AG1IA_01604**	yes	no	211	2	n.a.	yes	DUF1524 domain containing-protein	15.46	18.00	14.69	16.05
**AG1IA_01605**	no	no	56	2	n.a.		hypothetical protein	148.22	164.07	160.04	157.44
**AG1IA_01933**	n,a,	n.a.	88	4	n.a.		hypothetical protein	0.91	2.65	2.15	1.90
AG1IA_02138	yes	yes	290>	n.a.	n.a.		hypothetical protein	58.78	74.98	67.94	67.23
AG1IA_02490 [f]	n.a.	n.a.	-	n.a.	n.a.		hypothetical protein	0.59	0.00	0.00	0.20
AG1IA_02878/79/80 [g]	yes	yes	759	n.a.	24 hpi		DNA binding domain-containing protein	35.98	47.58	45.14	42.90
**AG1IA_02940**	yes	no	209	6	n.a.		DUF3455 domain containing-protein	430.26	251.35	207.44	296.35
**AG1IA_03047**	n.a.	n.a.	68	5	n.a.		hypothetical protein	1.30	0.73	0.00	0.67
**AG1IA_03254**	no	no	111	3 or 6	10 hpi		hypothetical protein	9.03	12.85	14.38	12.09
**AG1IA_04006**	no	no	54	2	n.a.		hypothetical protein	8.63	21.74	13.00	14.46
AG1IA_04108	yes	yes	52	n.a.	24 hpi		hypothetical protein	2.12	4.91	6.18	4.40
AG1IA_04191	yes	yes	98	n.a.	10 hpi		hypothetical protein	0.62	1.00	0.16	0.59
**AG1IA_04237**	yes	no	138	5	24 hpi		hypothetical protein	0.49	0.52	0.12	0.38

AG1IA_04267/8 [g]	yes	yes	478	n.a.	n.a.		Viral tegment protein/pectinesterase inhibitor	53.65	55.73	54.81	54.73
**AG1IA_04298**	yes	no	218	4	n.a.		emp24/gp25L/p24 family/GOLD domain-containing protein	66.30	64.66	71.39	67.45
**AG1IA_04737**	n.a.	n.a.	140	n.d.	n.a.		hypothetical protein	0.00	0.00	0.00	0.00
**AG1IA_04814**	n.a.	n.a.	145	n.d.	10 hpi		hypothetical protein	0.22	0.36	0.23	0.27
**AG1IA_04819**	no	no	151	5	n.a.		fungal cellulose binding domain-containing protein	0.00	0.00	0.00	0.00
AG1IA_04889 [f]	n.a.	n.a.	-	n.a.	n.a.		hypothetical protein	0.00	0.00	0.00	0.00
AG1IA_05179	yes	yes	60	n.a.	n.a.	yes	hypothetical protein	0.13	0.29	0.00	0.14
**AG1IA_05291**	no	no	230	3	24 hpi	yes	hypothetical protein	35.87	41.33	33.47	36.89
**AG1IA_05500**	yes	no	227>	2	n.a.		hypothetical protein	780.36	928.89	845.63	851.63
AG1IA_05601 [f]	n.a.	n.a.	-	n.a.	24 hpi		histone acetyl transferase	15.28	11.79	19.81	15.63
**AG1IA_05783**	no	no	90	5	n.a.		hypothetical protein	27.82	56.66	58.08	47.52
**AG1IA_05938**	yes	no	189	1	n.a.		hypothetical protein	28.44	78.85	63.30	56.86
**AG1IA_06169**	n.a.	n.a.	184	n.d.	24 hpi		hypothetical protein	0.00	0.00	0.00	0.00
**AG1IA_06325**	no	no	142	2	n.a.	yes	FKBP-type peptidyl-prolyl cis-trans isomerase domain-containing protein	149.73	212.66	194.36	185.58
**AG1IA_06613** [h]	yes	no	218	2	n.a.	yes	DUF3129 domain containing-protein	183.12	312.28	273.76	256.39
**AG1IA_06710** [h]	yes	no	38	6	n.a.		hypothetical protein	0.00	0.63	0.40	0.34
**AG1IA_06739**	yes	no	221	4	18, 24 hpi		hypothetical protein	9.26	7.92	9.28	8.82
AG1IA_06741	yes	yes	59	n.a.	18, 24 hpi		hypothetical protein	1.26	0.00	1.68	0.98
**AG1IA_07047**	yes	no	154	6	24 hpi		hypothetical protein	31.69	30.05	25.03	28.92
**AG1IA_07075**	yes	no	117	5	n.a.	yes	CFEM domain-containing protein	1.57	4.68	3.22	3.15
**AG1IA_07122**	n.a.	n.a.	133	5	n.a.		hypothetical protein	0.00	0.00	0.00	0.00
**AG1IA_07262**	n.a.	n.a.	257	n.d.	n.a.	yes	hypothetical protein	0.25	0.10	0.20	0.18
**AG1IA_07267** [h]	yes	no	159	1	n.a.	yes	hypothetical protein	5.80	6.46	8.31	6.86
AG1IA_07490	yes	yes	129	n.a.	n.a.		Yippee domain-containing protein	10.59	10.34	12.10	11.01
**AG1IA_07958** [h]	yes	no	306	4	18, 24 hpi		igA peptidase M64 domain-containing protein	13.85	10.61	12.07	12.18
**AG1IA_07966**	yes	no	142	1	10 hpi		hypothetical protein	8.19	9.09	9.30	8.86
**AG1IA_08025**	no	no	238	2	n.a.		DUF3421 domain containing-protein	38.43	44.08	37.76	40.09
**AG1IA_08061**	n.a.	n.a.	184	5	10, 18 hpi		hypothetical protein	0.23	0.57	0.08	0.30
**AG1IA_08487** [h]	yes	no	144	3 or 6	24 hpi		Ribonuclease domain-containing protein	384.71	416.22	430.21	410.38
**AG1IA_08488**	yes	no	149	2	24 hpi	yes	Ribonuclease domain-containing protein	2.28	3.45	0.98	2.24
**AG1IA_08577**	n.a.	n.a.	49	n.d.	n.a.		hypothetical protein	0.00	0.00	0.00	0.00
**AG1IA_08615**	yes	no	225	1	10 hpi	yes	hypothetical protein	24.78	25.15	25.99	25.31
**AG1IA_08650**	yes	no	213>	5 or 6	24 hpi		hypothetical protein	23.90	58.04	48.62	43.52
**AG1IA_08777**	yes	no	160	6	24 hpi		plastocyanin-like domain-containing protein	0.48	0.72	0.77	0.65
AG1IA_08781 [f]	n.a.	n.a.	-	n.a.	18, 24 hpi		Cuticle-degrading protease	61.13	42.02	19.48	40.87

AG1IA_08891	yes	yes	147	n.a.	n.a.	yes	hypothetical protein	59.57	47.90	41.50	49.65
AG1IA_08892	yes	yes	169	n.a.	n.a.	yes	hypothetical protein	63.82	47.94	45.15	52.30
AG1IA_08957	yes	yes	32	n.a.	n.a.		hypothetical protein	0.00	0.00	0.00	0.00
**AG1IA_09055**	yes	no	265	5	n.a.	yes	thaumatin-like protein	2.67	2.82	2.26	2.58
**AG1IA_09060**	yes	no	253	4	n.a.	yes	thaumatin-like protein	5.50	6.78	5.84	6.04
AG1IA_09137 [f]	n.a.	n.a.	-	n.a.	n.a.		hypothetical protein	5.06	2.22	3.63	3.64
**AG1IA_09202**	yes	no	184	2	n.a.	yes	hypothetical protein	106.32	205.36	185.35	165.68
**AG1IA_09203**	yes	no	181	2	n.a.	yes	hypothetical protein	38.83	52.21	43.66	44.90
**AG1IA_09207**	yes	no	182	1	24 hpi	yes	hypothetical protein	316.59	322.34	235.00	291.31
**AG1IA_09229**	yes	no	99	4	n.a.		hypothetical protein	2.50	1.32	1.04	1.62
AG1IA_09275	yes	yes	158	n.a.	n.a.		hypothetical protein	521.10	414.59	355.22	430.30
**AG1IA_09643**	n.a.	n.a.	66	n.d.	n.a.		hypothetical protein	0.00	0.00	0.00	0.00
**AG1IA_09728**	yes	no	67	5	n.a.		hypothetical protein	1.24	1.54	0.74	1.18
AG1IA_09836	yes	yes	147	n.a.	18 hpi		hypothetical protein	47.64	41.95	36.13	41.91
**AG1IA_09837**	n.a.	n.a.	131	n.d.	10 hpi		hypothetical protein	0.00	0.00	0.00	0.00
**AG1IA_09956**	no	no	84	3 or 4	n.a.	yes	hypothetical protein	173.67	148.51	123.75	148.64
**AG1IA_10146**	yes	no	82	4 or 6	n.a.		hypothetical protein	38.00	98.71	86.25	74.32
AG1IA_10180	yes	yes	146	n.a.	n.a.		hypothetical protein	2.23	2.63	3.06	2.64
**AG1IA_10284**	yes	no	80>	1	n.a.		hypothetical protein	0.00	0.00	0.00	0.00
AG1IA_10296 [f]	n.a.	n.a.	-	n.a.	10 hpi		hypothetical protein	24.79	20.71	24.11	23.20
AG1IA_10375	yes	yes	168>	n.a.	n.a.		valine-tRNA ligase	72.02	72.31	64.21	69.52
AG1IA_10403	yes	yes	152>	n.a.	n.a.		hypothetical protein	9.10	8.83	12.65	10.19
AG1IA_10405	yes	yes	65>	n.a.	18, 24 hpi	yes	hypothetical protein	0.40	0.45	0.81	0.55
**AG1IA_10478**	no	no	93	5	n.a.		hypothetical protein	12.03	14.08	17.09	14.40

[a] Finally selected *RsSEPGs* after verification of gene model were shown in bold letter.

[b] Cluster numbers categorized with their expression profiles during infection on *Brachypodium distachyon* in this study as shown in Fig. 2.

[c] Expression timing (hours) after inoculation on rice plants determined by Zheng et al. (2013).

[d] Annotations were obtained from top hit of blastP search.

[e] FPKM (fragments per kilobase of exon per million reads mapped) of 3 independent RNA-seq analysis using total RNA prepared from growing hyphae of *R. solani*.

[f] Transcripts were detected in reverse strand of these genes in RNA-seq results.

[g] A series of transcript was detected across multiple annotated genes.

[h] Prediction as an effector protein with EffectorP 1.0 was lost in the calibrated cDNA sequences of these 6 genes.

n.d., not detected

n.a., not applicable

Supplementary Table S4. Primers used in this study

Gene	Primer sequence	Gene	Primer sequence
AG1IA_00157	CCCATGCTCACTTGGCTTC<br>CCCTTACTTCGTCATCATACTCACC	*AG1IA_07122*	TCTCTAGTCAGCCGCCCATC<br>GATTTGTTCTCTGGTTTCGAGGTC
AG1IA_00273	CCGCAATGCAAGTCAAAGG<br>GCAAAGGGACGAAGGTGTG	*AG1IA_07262*	TTGATCTTGTGCTCTGCCATC<br>CTTTCCTCCGCCATTACACC
AG1IA_00431	GGGATCGACTGGGATAAAAGTG<br>ATCGCACCTTGCCGTTG	*AG1IA_07267*	GTCGACTGGCTCACACCTTC<br>ACGCACTAAACAGCGCAAAC
AG1IA_00478	GGGTTGACGAGTTTGCTTCC<br>TAATAGCGTGGTCCGTTCTTCC	*AG1IA_07958*	GCGATCAATATCAACGACCAAG<br>TGAGAGGGAGTATGACGGAGTG
AG1IA_00669	TGTTCTCTTCGCTTGTATCGTTTG<br>ACGACGGATTGGAGACTTGG	*AG1IA_07966*	GATCCATGCTATGCCAGTACCTC<br>GTCGCCATCCACAACCATC
AG1IA_00684	GGCAAGTTCCAAGGGAGTG<br>AAATCCAACGACCGGAGAAG	*AG1IA_08025*	GACTCTTGCAACCACAGTCTCC<br>TTCCTCTGCCTTTCGTATCC
AG1IA_00763	TGGGAGAAAGGGACAGCAAG<br>ACAGTGATTTGCCTCGTTCG	*AG1IA_08061*	GGTCGCAGTTTCATCATCTCC<br>TTGCGGCGAATCCAAAG
AG1IA_00879	TGACTGAGATGGAGGAAATCACAC<br>TTCCTGGTAGCCTGTAATCGTAATC	*AG1IA_08487*	CACGTTTTCAATAACAGGGAAGG<br>TAAACCGACGAGCGGAAAAG
AG1IA_00951	GGCCGATGTCGAGAACTTG<br>TACACCGCGAGGGTCTTTG	*AG1IA_08488*	CTTTGTTCGAGTTCCCTATTTTCC<br>AGCCCCTTATACTGCCGATG
AG1IA_01042	CATCTGGGATTGACGCTTATGTC<br>TTCCTGCTGCCGATTTCC	*AG1IA_08577*	CTTGCTTGTGCCTAATACGACTG<br>TTTGACGGGGTGGCTTG
AG1IA_01512	GGCCTCAGGAAGACACAAGG<br>TGGAAGGGATAGGTGGGAAAC	*AG1IA_08615*	CCCTCTTGTAATCAACGTCTTGG<br>CTACTGCCTCCTTGTTCATCTGG
AG1IA_01603	CTAAATTGGTTGTTGCTG<br>CGCTCTCGTGTATCGCATTTAC	*AG1IA_08650*	TTTACGACCAACCCGCCTAC<br>CCTGGTTAAGTCCAGCCAACTC
AG1IA_01604	CGAAGGCAGATGCGATACAC<br>TCATAGTCCGAAGTCCAAAGTCC	*AG1IA_08777*	CCAAGGAATCAGGAGGAAAGTG<br>TCCAAAAGGGTCCGTGTAATG
AG1IA_01605	CGCTTCGCTTATTTGATTGCTC<br>GGGTCGGTTGTCGCTTTAC	*AG1IA_09055*	TGCGAATGTAGTCGCTCACC<br>TAAACCCGCCAACCAAACTC
AG1IA_01933	AGGCACCGGCTAATTGTTG<br>CCAGACGATCCCAAATCAACTC	*AG1IA_09060*	CGCCTGTCCATTCACCATC<br>CCAAATACGACCAGCCTTCC
AG1IA_02940	AACACATCGCTCTTGGATATGG<br>TTCTGGGTCGGTTTTGGTG	*AG1IA_09202*	AGGCTTTCTGCTCGCCTTC<br>GTTGTCCACTCCCTCCACTACC
AG1IA_03047	GACCATTTCCAAGACGACCAG<br>GGGAATCGGGATAATGCAAG	*AG1IA_09203*	GCCTTTGCTTCCCCTCTTG<br>GTAAGATTGCATTTGCCCTTCC
AG1IA_03254	GAAAGGCACGGATAGGAAAGG<br>GCCGAGTACGGTAACAAAAGG	*AG1IA_09207*	GTGGAATGCGCGTGTACTG<br>CGTATTTTCCGCCCTTCTTG
AG1IA_04006	TTCGTCCGGCTTGTTCG<br>ACACGGGGTGAAGAGACAGG	*AG1IA_09229*	GGCTCCACCCAGAGCATAAATAC<br>CGCATTACCTCCTCGCCTAC
AG1IA_04237	GCCCCAGTATCTCTGTTTTGTTG<br>CACAATGACCTCCAAAATTCTTCC	*AG1IA_09643*	TTTTGTGATTCTGGCCTGTTGTAG<br>CAGACCGCACAGGTCCTAAC
AG1IA_04298	GTCGTAGACAGATCAGAACACCAAC<br>CGAAATAACACTCCCCACCAAG	*AG1IA_09728*	TGGCCGGACCAACAATAAG<br>CGAGAAAGCGTCATAGAAACAATC
AG1IA_04737	AGCGTGGTGAAGCGAATG<br>CAGACGGATGGCTGTAACGTAG	*AG1IA_09837*	AAGGTGGTGGGCAGTAACG<br>GGATGAACAACACCCTGATCC
AG1IA_04814	CATAGGAAACGCTCGGCAAC<br>TGGCACTGATGAGACGGTAGAG	*AG1IA_09956*	TAGCAGAGATTGCTGCACAAGAG<br>ACGGCAACACTCCTTGTATCTTC
AG1IA_04819	CCGTCAGTTTCGTGTAATCACTATG<br>AGATGTGATTGTGGTGGTTGATG	*AG1IA_10146*	TTTCGTCGCTGCCCAAG<br>CCTGGAAGAGGTGAGCCAAG
AG1IA_05291	GCGCGGCTGTTTTCTTG<br>GACACGAGAGTTGGCGAATG	*AG1IA_10284*	ATCCGCCACCTCTGTTAGC<br>CAGGTGCTGGCGATACAAC
AG1IA_05500	GGACTGCTGCATTAAGGTTGG<br>GCACGATGGCTGGTTGTAAG	*AG1IA_10478*	CGACTCCCTTGCCTATTTCTTG<br>CAAGTTTCTCCACTCCCTACGAAC
AG1IA_05783	CCGAGCCGAGAAGATCAAG<br>AGTTATTGGTCGAAGTCAGGGAAG	*18S rRNA*	AATTCCAGCTCCAATAGCGT<br>TACATACCGTGAGGCAGACC
AG1IA_05938	CAAGAGCAAACGACCGATG<br>GCCAAGGTCCAACAGCAAG		
AG1IA_06169	GACAATCAGGACGACAACGAG<br>ATTGCAGACTTTAGCGAACCAAG	*AG1IA_05310*	CAACAAGAAAAGTTCACCGAGTATG<br>CGACTTTCGGTTCGTTTGTG
AG1IA_06325	GTGTGTTGGTGAAAAGCGAAAG<br>GGTCCTTGATGCCCAATAGTTC	*AG1IA_06618*	GCGGGGTTAGTTGTGGTTG<br>GGCTTCATAGCAGGGGAAAAG
AG1IA_06613	TTGTTGTCGTTGCTGCTCTTG<br>CTGCCTTTCCGTTGATACCC	*AG1IA_06890*	ACCAGGACCACGACAAGGAC<br>TGCTATCCGAGTGCCCAAC
AG1IA_06710	GATACGGCTCGAAGCAACTAAG<br>GGTTTTACTCGCCACTGCTTG	*AG1IA_07787*	TCCCTTTTCGTTCAGTGTAGTTAGG<br>TTATTGGGCAGGATCGTTTTG
AG1IA_06739	CGCCTCTGGTGGTTCTATCTACTC<br>GAAATCCGAAATGATTGCCTTG	*AG1IA_07795*	GGATCAACTGGGCAACTCAAC<br>GCAGCTACTACAACATGCACACC
AG1IA_07047	GCGTTGCTATCTGCGTTCC<br>GTAGTCTTCCCGTCCCATCC	*AG1IA_08771*	AGACCAGTACGCCTTCACCAC<br>CCCGATAAGCAACCAGAACC
AG1IA_07075	CCGATTGCCGTTGGAAG<br>AGTAGAATACACCTGGGCGAAAAC	*AG1IA_09161*	GCCCATACGCTCTTCCAAAC<br>CACCCAACAGCGACTTCATC
